# Physical disability and risk of incident hypertension: a prospective cohort analysis

**DOI:** 10.1038/s41371-025-01061-1

**Published:** 2025-08-22

**Authors:** Yusuff Adebayo Adebisi, Najim Z. Alshahrani, Isaac Olushola Ogunkola, Don Eliseo Lucero-Prisno

**Affiliations:** 1 College of Social Sciences, University of Glasgow, Glasgow, UK; 2 Scottish Centre for Administrative Data Research, University of Edinburgh, Edinburgh, UK; 3 Department of Family and Community Medicine, Faculty of Medicine, University of Jeddah, Jeddah, Saudi Arabia; 4 Nuffield Department of Population Health, University of Oxford, Oxford, UK; 5 Department of Global Health and Development, London School of Hygiene and Tropical Medicine, London, UK

**Keywords:** Risk factors, Preventive medicine

## Abstract

Hypertension remains a leading cause of morbidity and mortality worldwide, yet its relationship with physical disability has been underexplored in population-based longitudinal studies. We conducted a prospective cohort analysis using data from the UK Household Longitudinal Study. Individuals aged 16 and older without baseline hypertension (*N* = 19,319) were followed from Wave 10 (2018–19) to Wave 14 (2022–23). Physical disability was defined as self-reported difficulty, lasting or expected to last at least 12 months, in any of eight domains of physical and sensory functioning. Modified Poisson regression with robust standard errors was used to estimate adjusted relative risks (RRs) for incident hypertension, controlling for age group, sex, residential location (urban/rural), educational attainment, ethnicity, smoking status, and baseline health conditions. Over a four-year follow-up, 610 of 19,319 participants (3.2%) developed hypertension. In fully adjusted model, individuals with any physical disability had a significantly elevated risk of hypertension (RR = 1.65; 95% CI:1.39–1.97; *p* < 0.001) compared to those without disability. Compared to individuals with no disability, those with one physical disability had an RR of 1.29 (95% CI:1.01–1.64; *p* = 0.043), while those with two or more disabilities had an RR of 2.10 (95% CI:1.69–2.59; *p* < 0.001). A linear trend was observed across increasing number of disabilities (RR per additional domain = 1.43; 95% CI:1.29–1.59; *p* < 0.001). By type, the highest risks were observed for coordination or balance impairment (RR = 2.12; 95% CI:1.58–2.84), mobility impairment (RR = 2.03; 95% CI:1.66–2.48), and sight impairment (RR = 1.80; 95% CI:1.27–2.56), all *p* < 0.001. Physical disability was a significant independent predictor of incident hypertension in this population-based cohort.

## Introduction

Hypertension is a leading cause of premature mortality and a major modifiable risk factor for cardiovascular disease globally [1, 2]. Despite widespread efforts to improve screening and control, the burden of hypertension remains substantial, particularly in older adults and socioeconomically disadvantaged populations [3, 4]. Understanding the social and functional determinants of hypertension risk is critical to developing more equitable and effective prevention strategies. While traditional risk factors such as age, obesity, diet, and smoking are well established [5, 6], there is growing recognition that non-biomedical factors, such as physical functioning and disability status, may also influence cardiovascular risk trajectories [7].

Physical disability, defined as limitations in mobility, dexterity, or self-care, affects a significant and growing proportion of adults in the UK and worldwide [8, 9]. Disability is associated with a range of adverse health outcomes, including increased risk of hospitalisation, poorer mental health, and reduced access to preventive care [10]. Individuals with physical disabilities often face structural barriers to healthy living, such as inaccessible environments, social exclusion, and under-diagnosis of chronic conditions [10]. Despite this, physical disability is rarely considered in hypertension risk assessments, and there is limited longitudinal evidence examining whether disability independently predicts future development of hypertension in the general population.

Existing studies exploring the relationship between disability and hypertension have several limitations. Many have been cross-sectional in design, preventing clear conclusions about temporality and causality [11–14]. Others have focused narrowly on specific disability types or have relied on clinical samples that do not reflect the broader population. Furthermore, few studies have assessed whether the number or type of physical impairments influences hypertension risk in a graded manner. As a result, it remains unclear whether physical disability serves as a distinct predictor of hypertension incidence, above and beyond established demographic, behavioural, and clinical risk factors. To address this gap, we conducted a prospective cohort analysis to examine whether physical disability at baseline predicts incident hypertension over a four-year period.

## Method

### Study design, data source and study population

This prospective cohort study used data from the UK Household Longitudinal Study (UKHLS), also known as Understanding Society, a nationally representative longitudinal panel survey that follows individuals aged 16 and over living in private households across the United Kingdom [12–14]. Initiated in 2009, UKHLS employs a clustered, stratified probability sampling design, including an ethnic minority boost sample to enhance representativeness and support subgroup analyses. Households were selected from the Postcode Address File using a multi-stage sampling approach, with stratification by region and demographic characteristics and clustering at the level of Primary Sampling Units (typically postcode sectors) [12]. Data are collected annually through face-to-face interviews, web-based surveys, and self-completion questionnaires, covering domains such as health, employment, income, education, and family dynamics [15].

For this study, Wave 10 (2018–19) served as the baseline and Wave 14 (2022–23) as the follow-up. Wave 10 included 34,319 adult respondents, and Wave 14 included 35,471. Of the 46,405 unique individuals who participated in either or both waves, 23,385 (50.4%) were successfully matched across Waves 10 and 14 using a unique personal identifier. Participants with missing data, proxy responses, refusals, or “don’t know” responses to the hypertension question in Wave 14 (*n* = 234), as well as those who reported high blood pressure at baseline (*n* = 3832), were excluded. After applying these criteria, the final analytic sample comprised 19,319 individuals aged 16 and above.

### Exposure: physical disability

The primary exposure was physical disability, defined using self-reported long-standing difficulty (lasting, or expected to last, at least 12 months) in any of eight functional domains recorded in Wave 10 of Understanding Society: mobility (moving around at home and walking), lifting/carrying objects, manual dexterity (using hands to carry out everyday tasks), continence (bladder and bowel control), physical coordination, personal care, hearing (apart from using a standard hearing aid), and sight (apart from wearing standard glasses) [16, 17]. Each domain was measured using a binary indicator. Respondents were coded as having a physical disability if they reported difficulty in at least one of these domains.

In addition to this binary indicator, two secondary measures of disability were constructed. First, we derived a disability count variable by summing the number of affected physical domains (range: 0–8), which was then categorised into three levels: no disabilities (0 domain), one domain affected, and two or more domains affected. This categorization was chosen based on the distribution of the data, and to ensure sufficient statistical power for reliable estimates in each category. Second, we generated a set of binary variables indicating the presence or absence of each specific physical disability domain. This allowed us to examine heterogeneity in hypertension risk by disability type.

The individual domains assessed reflect specific physical and sensory impairments that contribute to broader functional limitations. While the UKHLS labels these measures under “disability,” they align with the impairment and activity limitation constructs in the ICF framework [16]. For consistency with the dataset and existing literature, we use the term physical disability throughout this study to refer to these self-reported limitations.

### Outcome: incident hypertension

The primary outcome was incident hypertension, defined as a new self-reported doctor diagnosis of high blood pressure between baseline (Wave 10) and follow-up (Wave 14). At Wave 14, participants were asked whether they had been newly diagnosed with high blood pressure or hypertension since their last interview. Respondents who answered “yes” were classified as having developed incident hypertension, while those who answered “no” were classified as non-cases. Participants with proxy interviews, refusals, or “don’t know” responses were treated as missing and excluded from the analytic sample. Previous research has shown that self-reported hypertension in population-based surveys demonstrates acceptable validity and reliability when compared with clinical measurements [18].

To ensure that only new cases of hypertension were captured, individuals who reported having high blood pressure at baseline (Wave 10) were excluded from the cohort. Baseline hypertension was identified using the variable which records whether the respondent had ever been told by a health professional that they had high blood pressure. This exclusion helped establish a clean prospective cohort for the analysis of hypertension incidence. The final outcome variable was coded as binary (1 = developed hypertension; 0 = remained normotensive), with a total of 610 incident cases identified over the four-year follow-up period.

### Covariates

Covariates were selected based on prior evidence of their associations with both physical disability and hypertension [19]. All covariates were measured at baseline (Wave 10). Age was categorised into four groups: 16–34, 35–49, 50–64, and 65 years or older. Sex was coded as male or female. Residential location was defined as urban or rural based on household classification. Educational attainment was grouped into three levels: degree or higher, upper secondary, and lower secondary or no qualifications. Ethnicity was categorised as White, Mixed, Asian, Black, or Other.

**Fig. 1:**
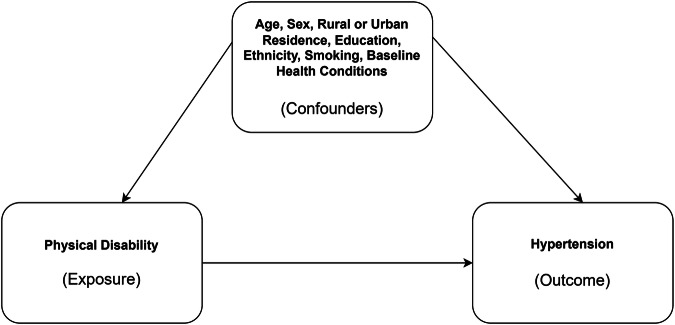
Directed acyclic graph illustrating baseline confounders of the association between physical disability and subsequent hypertension.

Smoking status was included as a binary indicator distinguishing current smokers from non-smokers. Baseline health status was captured using a binary variable indicating the presence of one or more self-reported longstanding health conditions, based on diagnoses recorded in the Wave 10 health modules. This included self-reported diagnoses of cardiovascular diseases (e.g. coronary heart disease, heart failure, myocardial infarction, angina, stroke), respiratory illnesses (e.g. chronic bronchitis, emphysema, COPD), endocrine and metabolic disorders (e.g. type 1 and type 2 diabetes, gestational diabetes, hypothyroidism), neurological conditions (e.g. epilepsy, multiple sclerosis), musculoskeletal disorders (e.g. rheumatoid arthritis, osteoporosis), cancers (e.g. bowel, lung, breast, prostate, skin, liver, and others), and psychiatric or emotional disorders (e.g. anxiety, depression, bipolar disorder, psychosis, PTSD, eating disorders). This adjustment aimed to account for underlying morbidity that could confound the association between physical disability and subsequent hypertension risk [See Fig. 1]. Participants with missing covariate data were retained by categorizing missing values as a separate group.

### Statistical analyses

Descriptive analyses were first conducted to summarise baseline characteristics of the study population by physical disability status. Categorical variables were presented as frequencies and percentages, and group differences were assessed using chi-square tests.

To estimate the association between physical disability and incident hypertension, we used modified Poisson regression with robust standard errors to calculate relative risks (RRs) and 95% confidence intervals (CIs) [20]. Modified Poisson regression was chosen to estimate relative risks for the binary outcome of incident hypertension, as it provides robust and consistent estimates even for common outcomes, unlike log-binomial regression which may fail to converge, and avoids the time-to-event assumptions of Cox proportional hazards models, which were less suitable given the fixed four-year follow-up and lack of precise event timing. Three sequential models were fitted. Model 1 was unadjusted and estimated the crude association between physical disability and incident hypertension. Model 2 adjusted for age group and sex. Model 3 was fully adjusted and included additional covariates: residential location (urban/rural), educational attainment (degree or higher, upper secondary, lower or none), ethnicity (White, Mixed, Asian, Black, Other), smoking status (current vs. not), and the presence of one or more longstanding health conditions at baseline (excluding hypertension). All covariates were defined based on Wave 10 responses.

Fully adjusted models were also used to examine associations between the number of physical disabilities and risk of incident hypertension. The disability count variable was categorised into three levels: none (reference), one domain, and two or more domains. A test for linear trend was conducted by including the count of disabilities as a continuous variable in the model. Polynomial contrasts were used to assess departures from linearity. Another fully adjusted models were also used to estimate the association between specific types of physical disability and incident hypertension. For each domain, a binary indicator variable was created reflecting the presence or absence of that particular limitation.

Analyses were conducted using Stata version 18. Two-sided *p*-values were used throughout, with values less than 0.05 considered statistically significant.

## Result

The final analytic sample comprised 19,319 adults, of whom 3316 (17.2%) reported at least one physical disability at baseline, while 16,003 (82.8%) reported none. Over the four-year follow-up, 610 participants (3.2%) developed incident hypertension.

Table 1 presents the baseline characteristics of the study population stratified by physical disability status. Respondents with physical disability were significantly older than those without; for example, 34.4% of those with disability were aged 65 and over compared to 15.5% of those without (*p* < 0.001). A higher proportion of women reported physical disability (62.2 vs. 56.4%, *p* < 0.001). No significant differences were observed in urban versus rural residence. Educational attainment differed markedly by disability status. Respondents with physical disability were less likely to hold a degree or higher qualification (28.5 vs. 39.2%) and more likely to have lower or no formal qualifications (48.2 vs. 37.1%; *p* < 0.001). Ethnic distribution also differed slightly, with a higher proportion of White respondents among those with physical disability (86.7 vs. 83.8%; *p* = 0.001). Current smoking was more common among individuals with physical disability (16.0 vs. 10.6%; *p* < 0.001), as was the presence of one or more baseline health conditions (64.1 vs. 30.1%; *p* < 0.001). Incident hypertension was significantly more frequent among respondents with physical disability (5.7%) compared to those without (2.6%; *p* < 0.001).

**Table 1:** Baseline characteristics of respondents by physical disability status.

Characteristics	No physical disability (*n* = 16,003)	Has physical disability (*n* = 3316)	All (*n* = 19,319)	*P*-value
**Age group, n (%)**				<0.001
16–34	4500 (28.1)	365 (11.0)	4865 (25.2)	
35–49	4697 (29.4)	701 (21.1)	5398 (27.9)	
50–64	4333 (27.1)	1110 (33.5)	5443 (28.2)	
65+	2473 (15.5)	1140 (34.4)	3613 (18.7)	
**Sex, n (%)**				<0.001
Male	6973 (43.6)	1254 (37.8)	8227 (42.6)	
Female	9030 (56.4)	2062 (62.2)	11,092 (57.4)	
**Residence, n (%)**				0.512
Urban	11,983 (74.9)	2465 (74.3)	14,448 (74.8)	
Rural	4020 (25.1)	851 (25.7)	4871 (25.2)	
**Education Level, n (%)**				<0.001
Degree or Higher	6278 (39.2)	946 (28.5)	7224 (37.4)	
Upper Secondary	1835 (11.5)	260 (7.8)	2095 (10.8)	
Lower or None	5943 (37.1)	1597 (48.2)	7540 (39.0)	
Missing	1947 (12.2)	513 (15.5)	2460 (12.7)	
**Ethnicity, n (%)**				0.001
White	13,417 (83.8)	2875 (86.7)	16,292 (84.3)	
Mixed	305 (1.9)	61 (1.8)	366 (1.9)	
Asian	1715 (10.7)	278 (8.4)	1993 (10.3)	
Black	461 (2.9)	80 (2.4)	541 (2.8)	
Other	105 (0.7)	22 (0.7)	127 (0.7)	
**Current Smoker, n (%)**				<0.001
Yes	1693 (10.6)	530 (16.0)	2223 (11.5)	
No	14,171 (88.6)	2763 (83.3)	16,934 (87.7)	
Missing	139 (0.8)	23 (0.7)	162 (0.8)	
**Baseline Health Condition, n (%)**				<0.001
Has condition(s)	4822 (30.1)	2126 (64.1)	6948 (36.0)	
No condition	10,596 (66.2)	1125 (33.9)	11,721 (60.7)	
Missing	585 (3.7)	65 (2.0)	650 (3.3)	
**Incident Hypertension, n (%)**				<0.001
No	15,583 (97.4)	3126 (94.3)	18,709 (96.8)	
Yes	420 (2.6)	190 (5.7)	610 (3.2)	

^*^P-values based on chi-square tests for categorical variables.

**Fig. 2:**
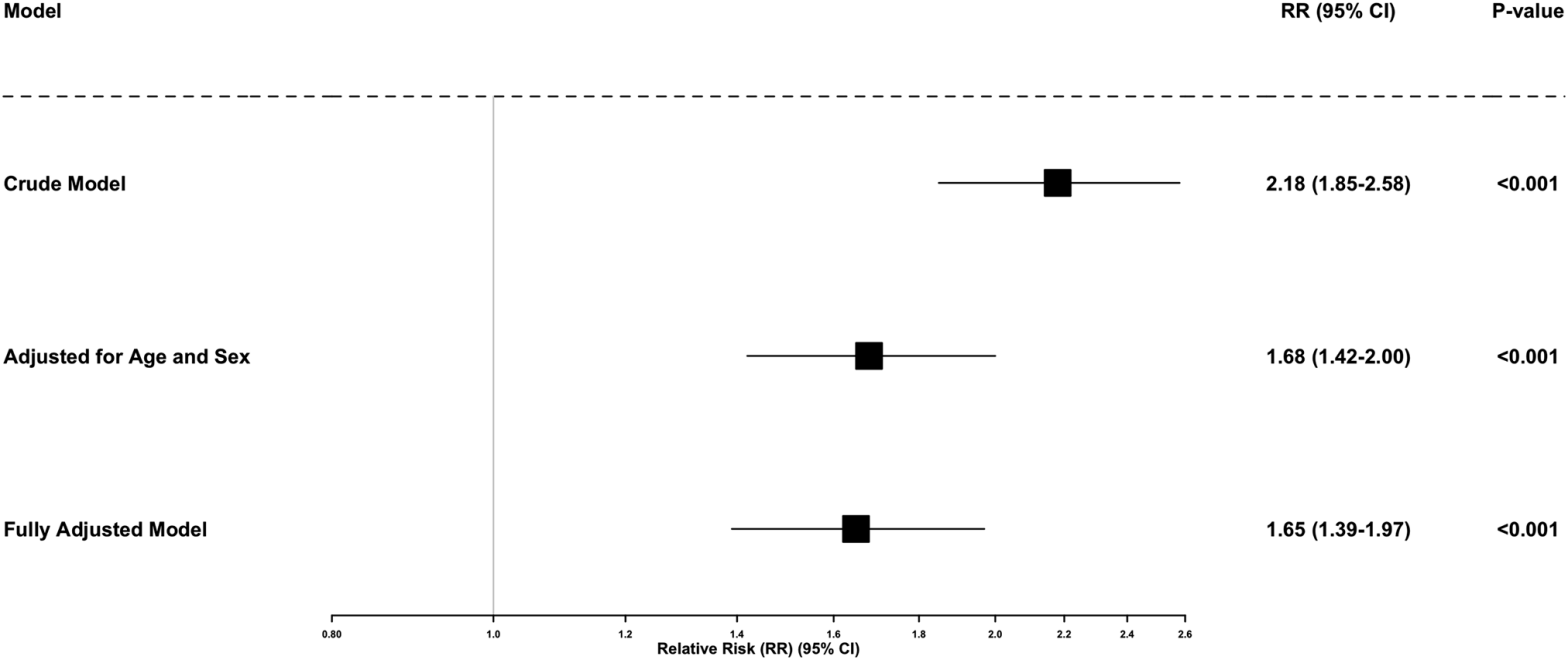
Crude and Adjusted Association between baseline physical disability and incident hypertension.

Table 2 and Fig. 2 display the results from modified Poisson regression models estimating the association between physical disability at baseline and incident hypertension at follow-up. In the unadjusted model (Model 1), individuals with physical disability had more than twice the risk of developing hypertension compared to those without disability (RR = 2.18; 95% CI: 1.85–2.58; *p* < 0.001). After adjusting for age group and sex (Model 2), the association remained strong, though attenuated (RR = 1.68; 95% CI: 1.42–2.00; *p* < 0.001). In the fully adjusted model (Model 3), which additionally controlled for residential location, educational attainment, ethnicity, smoking status, and baseline health conditions, physical disability was still associated with a significantly elevated risk of incident hypertension (RR = 1.65; 95% CI: 1.39–1.97; *p* < 0.001).

**Table 2:** Crude and adjusted relative risks (RRs) for incident hypertension at follow-up by physical disability status.

Model	Relative Risk (RR) 95% CI, *p*-value
Model 1: Crude	
No physical disability	Reference
Physical disability	2.18 (1.85–2.58), *p* < 0.001
Model 2: Adjusted for age and sex	
No physical disability	Reference
Physical disability	1.68 (1.42–2.00), *p* < 0.001
Model 3: Fully adjusted (Final) + Residence, Education, Ethnicity, Smoking, Baseline Health Conditions	
No physical disability	Reference
Physical disability	1.65 (1.39–1.97), *p* < 0.001

Table 3 presents adjusted relative risks for incident hypertension according to the number of physical disabilities reported at baseline. Compared to individuals without any physical disability, those reporting a single domain of physical disability had a significantly elevated risk of developing hypertension (RR = 1.29; 95% CI: 1.01–1.64; *p* = 0.043). The risk was substantially higher among individuals with two or more physical disabilities (RR = 2.10; 95% CI: 1.69–2.59; *p* < 0.001). A test for linear trend indicated a significant dose–response relationship between increasing number of disabilities and hypertension risk (RR per additional domain = 1.43; 95% CI: 1.29–1.59; *p* < 0.001). Polynomial contrast tests supported the linearity of this association (χ² = 46.03, *p* < 0.001), with no evidence of a quadratic (non-linear) trend (χ² = 0.91, *p* = 0.341).

**Table 3:** Adjusted relative risks (RRs) for incident hypertension by number of physical disabilities.

Number of Physical Disability	N	*Adjusted Relative Risk (RR) 95% CI, *P*-value
None	16,003	Reference
One	1764	1.29 (1.01–1.64), *p* = 0.043
Two or more	1552	2.10 (1.69–2.59), *p* < 0.001
**P-trend (Linear, continuous)**	–	1.43 (1.29–1.59), *p* < 0.001
Polynomial contrast	χ²	*P*-Value
Linear	46.03	*P* < 0.001
Quadratic	0.91	*P* = 0.341

^*^Model adjusted for age group, sex, urban or rural residence, educational attainment, ethnicity, smoking status, and baseline health conditions.

*RR* relative risk estimated using modified Poisson regression with robust standard errors.

**Fig. 3:**
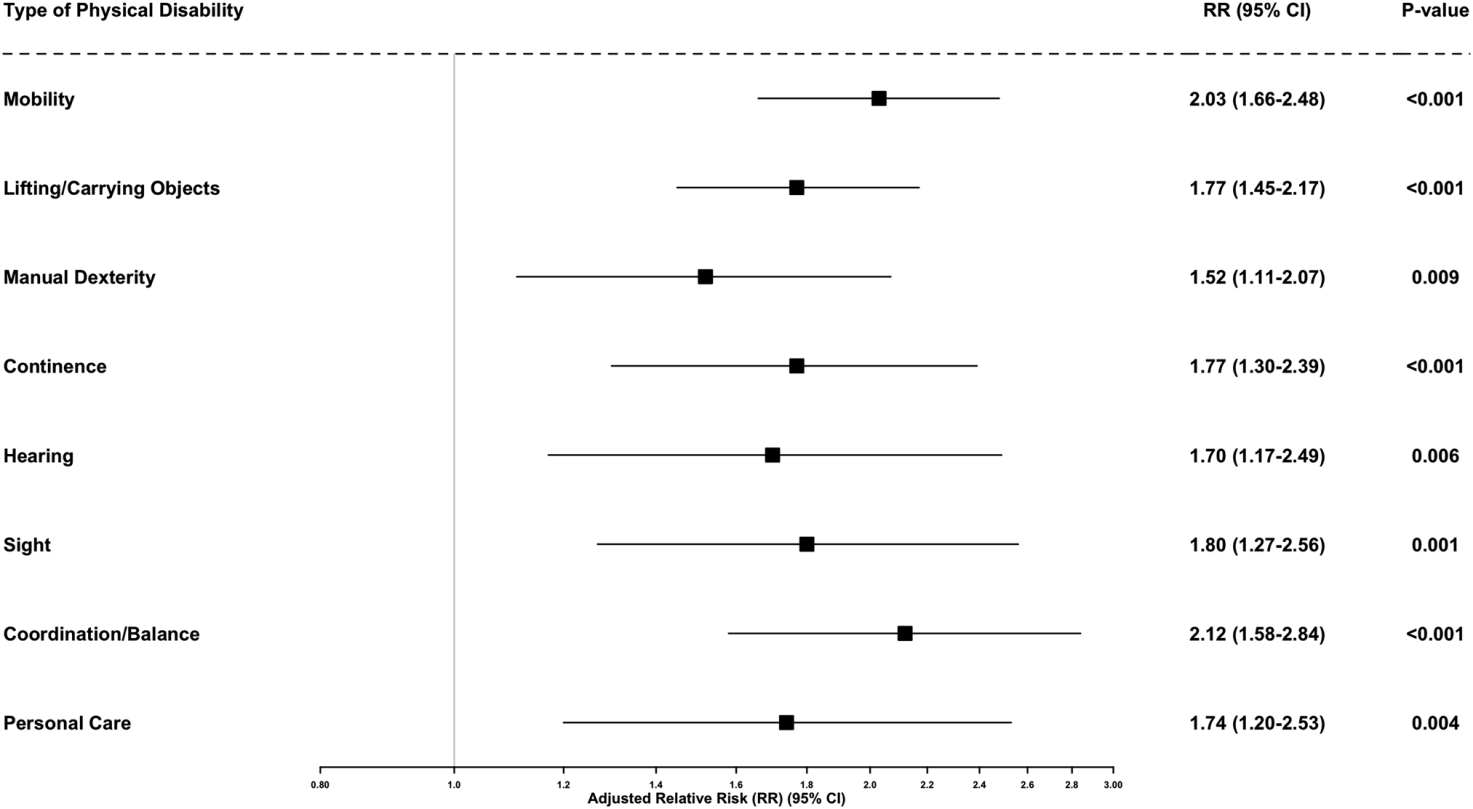
Adjusted Relative Risks (RRs) for Incident Hypertension by Type of Physical Disability.

Figure 3 presents the adjusted relative risks of incident hypertension associated with specific types of physical disability. All models controlled for age group, sex, residence, educational attainment, ethnicity, smoking status, and baseline health conditions. Across all domains, the presence of a specific physical limitation was significantly associated with increased risk of developing hypertension. The strongest associations were observed for coordination or balance impairments (RR = 2.12; 95% CI: 1.58–2.84; *p* < 0.001) and mobility impairments (RR = 2.03; 95% CI: 1.66–2.48; *p* < 0.001). Other limitations, including lifting or carrying objects (RR = 1.77; 95% CI: 1.45–2.17), continence problems (RR = 1.77; 95% CI: 1.30–2.39), and sight difficulties (RR = 1.80; 95% CI: 1.27–2.56), also showed substantial elevations in risk. Manual dexterity (RR = 1.52; 95% CI: 1.11–2.07), hearing (RR = 1.70; 95% CI: 1.17–2.49), and personal care limitations (RR = 1.74; 95% CI: 1.20–2.53) were similarly associated with elevated hypertension risk.

## Discussion

This study provides evidence that physical disability is an independent predictor of incident hypertension in a nationally representative UK adult population. Over a four-year follow-up, adults with physical disabilities at baseline were significantly more likely to develop hypertension than those without disabilities, even after accounting for a wide range of confounders. The risk was especially elevated among individuals with multiple impairments, with those reporting two or more functional limitations experiencing more than twice the risk of developing hypertension compared to individuals without disability. A clear linear trend was observed across disability count categories, suggesting that hypertension risk increases proportionally with the cumulative burden of physical disability. These results persisted in fully adjusted models, indicating that the observed associations are not fully explained by differences in age, sex, education, ethnicity, smoking, residential context, or existing health conditions. Together, these findings underline the importance of considering disability status as a key determinant of future cardiovascular risk.

This study makes several important contributions to the existing literature on disability and cardiometabolic health. While prior research has identified associations between functional limitations and hypertension, most existing studies have been cross-sectional, based on small or clinical samples, or restricted to single impairment types such as mobility or sensory loss [11–14]. Our findings extend this evidence by demonstrating, in a large and prospective cohort, that the relationship between physical disability and hypertension is both robust and generalisable. Importantly, we examined domain-specific associations and found elevated hypertension risks not only for mobility and coordination difficulties, which have been more widely studied, but also for less commonly assessed limitations such as continence, personal care, and manual dexterity. The consistently elevated relative risks across all disability types reinforce the idea that physical disability, in its various forms, contributes meaningfully to the development of hypertension, independent of shared risk factors. By taking a comprehensive and granular approach to the measurement of disability, our study clarifies that it is not merely the presence of a particular diagnosis but the broader experience of functional impairment that increases susceptibility to hypertension.

Several plausible mechanisms may help to explain the observed association between physical disability and incident hypertension. First, individuals with physical disabilities are more likely to experience reduced physical activity and prolonged sedentary behaviour [21, 22], both of which are known contributors to elevated blood pressure and vascular dysfunction [23–25]. Second, disability is often accompanied by higher levels of psychosocial stress, social isolation, and reduced access to preventive care [26, 27], which can all increase cardiovascular risk [28]. Third, some types of disability may reflect underlying neurological or musculoskeletal disorders associated with autonomic dysregulation or systemic inflammation, which have been linked to hypertension pathogenesis [29, 30]. For example, impairments in coordination or mobility may involve central nervous system pathways that also influence blood pressure control [31, 32]. Our findings of stronger associations in these domains are consistent with this hypothesis and align with prior studies suggesting that neurogenic and mobility-related impairments carry a disproportionately high cardiometabolic burden [33, 34]. Additionally, individuals with disability may face structural barriers to healthcare access and experience delayed diagnosis or under-treatment of hypertension, which may further exacerbate risk over time [35]. These interrelated pathways suggest that physical disability is not only a proxy for existing health burdens but also a source of physiological and social strain that increases future disease vulnerability.

This study also has practical implications for clinical care and public health. First, healthcare providers should consider physical disability as a key risk factor when assessing individuals for hypertension prevention and screening. Targeted interventions, including tailored advice on diet, exercise, and blood pressure monitoring, may be particularly beneficial in this population. Second, disability-inclusive approaches to cardiovascular risk management should be integrated into routine primary care, with specific attention to identifying and addressing barriers to care among individuals with functional limitations. Policies that enhance access to preventive services and promote inclusive environments may also help reduce cardiovascular disparities faced by disabled populations. Finally, our findings support the need for a broader public health framing of disability, not only as a demographic or social category but also as a clinical indicator of heightened chronic disease risk. Recognising and acting on this association could yield substantial gains in hypertension prevention and health equity.

While this study’s use of a nationally representative UK cohort enhances its generalizability within the UK context, its applicability to other populations warrants consideration. Differences in healthcare systems, disability prevalence, and hypertension screening practices globally may influence the observed association between physical disability and incident hypertension. For instance, in countries with less accessible healthcare systems, underdiagnosis of hypertension among individuals with disabilities due to limited access to care may weaken the observed association, whereas in settings with robust screening, the association may be stronger due to higher detection rates. Cultural differences in disability reporting, such as varying stigma or awareness of functional limitations, could also affect disability prevalence and the strength of its association with hypertension. Additionally, global variations in hypertension prevalence, driven by genetic, environmental, or lifestyle factors, may modify the relationship. These factors suggest that while our findings are robust within the UK, their generalizability to other populations requires further research in diverse settings.

Despite its strengths, this study has several limitations that should be considered when interpreting the results. First, physical disability was based on self-reported difficulties in functional domains, which may be influenced by individual perception, recall bias, or underreporting, particularly among marginalised groups. Nonetheless, the UKHLS disability measures align with the International Classification of Functioning, Disability and Health (ICF) framework [16, 17] and are widely used in population-based research, demonstrating acceptable reliability in large-scale surveys [36]. Second, although we adjusted for multiple known confounders, residual confounding by unmeasured factors such as physical activity levels, dietary habits, medication use (e.g., anti-inflammatory drugs or analgesics), or unreported comorbidities may influence the observed associations. Third, hypertension was based on self-reported physician diagnoses rather than clinical measurements, which may underestimate true incidence; however, this misclassification is unlikely to differ systematically by disability status and would likely bias estimates toward the null.

Furthermore, reverse causation remains a possibility, as undiagnosed or subclinical hypertension at baseline could contribute to physical disability through mechanisms such as microvascular damage or fatigue, potentially inflating the observed associations. While we excluded participants with diagnosed hypertension at baseline to mitigate this, undetected cases could still affect our findings. These limitations suggest that, while our study establishes a robust association between physical disability and incident hypertension, attributing causality requires further investigation with more detailed measurements of these factors and longer follow-up to confirm temporal relationships.

This study demonstrates that physical disability is a strong and independent predictor of incident hypertension in a nationally representative UK cohort. These findings highlight the need to recognise physical disability as a clinical risk factor in hypertension prevention and management. Incorporating disability-inclusive approaches into routine care and public health policy could help reduce cardiovascular health disparities and promote equitable outcomes.

## Summary

### What is known about this topic

- Physical disability is associated with poorer health outcomes, but its role in predicting incident hypertension has been understudied.

### What this study adds

- This study provides novel longitudinal evidence from a nationally representative UK cohort, demonstrating that baseline physical disability independently predicts incident hypertension over four years.
- A linear dose-response relationship was observed, with hypertension risk increasing with the number of physical disabilities.
- All physical disability domains were associated with increased hypertension risk, with notably strong associations for mobility and coordination difficulties.
- These findings highlight the need to include physical disability status in cardiovascular risk stratification and to adopt disability-inclusive prevention strategies.

## Data Availability

The data analysed in the study is publicly available University of Essex, Institute for Social and Economic Research. (2024). Understanding Society. [data series]. 13th Release. UK Data Service. SN: 2000053, 10.5255/UKDA-Series-2000053.
